# Prognostic Factors for Japanese Adults With Acute Community-Acquired Bacterial Meningitis: A Retrospective Study

**DOI:** 10.7759/cureus.57642

**Published:** 2024-04-05

**Authors:** Yoshiyuki Matsuki, Toshimi Oda, Eri Fukao, Ayaka Sugiura, Takayuki Yokozawa, Yutaka Honma

**Affiliations:** 1 Neurology, Showa General Hospital, Tokyo, JPN; 2 Infectious Diseases, Showa General Hospital, Tokyo, JPN; 3 Data Analysis, Deloitte Analytics, Tokyo, JPN; 4 Clinical Laboratory, Showa General Hospital, Tokyo, JPN

**Keywords:** retrospective study, cerebrospinal fluid cell count, japanese, neurologic prognosis, acute bacterial meningitis

## Abstract

Background

This study aimed to determine if the cerebrospinal fluid (CSF) cell count is useful for predicting the infection severity or prognosis in Japanese adults with community-acquired bacterial meningitis.

Methodology

This study retrospectively evaluated the prognosis of patients diagnosed with community-acquired bacterial meningitis at our hospital from January 2004 to December 2021 using the modified Rankin scale (mRs) (Showa General Hospital; N = 39). Patients were classified into the following two groups: (i) favorable (mRs: 0-3) and (ii) unfavorable (mRs: 4-6). Eight factors were selected and compared with outcomes, and then two factors were evaluated from those, and a multivariate logistic regression was used to determine the significant variables.

Results

CSF cell count was observed to be associated with poor prognoses (odds ratio (OR) = 0.86, 95% confidence interval (CI) = 0.99995-0.99999, p = 0.0012). Glasgow coma scale (GCS) score on admission was also observed to be associated with poor prognoses (OR = 0.93, 95% CI = 0.89145-0.97290, p = 0.0029).

Conclusions

Low CSF cell count and low GCS on admission were observed as risk factors for poor prognoses in patients with bacterial meningitis.

## Introduction

Bacterial meningitis is a neurological emergency disease and an inflammatory disease of the leptomeninges, which are the tissues surrounding the brain and spinal cord. It is characterized by bacterial infection of the subarachnoid space and cerebral ventricles, with an abnormal number of leukocytes in the cerebrospinal fluid (CSF) in most cases [1]. The mortality rate of bacterial meningitis remains high despite the appropriate use of effective antibiotics, and bacterial meningitis easily results in sequelae. A 2016 study reported that bacterial meningitis caused 318,000 deaths and 21,866,000 disability-adjusted life years annually worldwide [2].

Vaccination against *Streptococcus pneumoniae* and *Haemophilus influenzae* type b has dramatically decreased the number of patients with bacterial meningitis both in developed and developing countries. Consequently, the possibility of encountering patients with bacterial meningitis is decreasing [3]. Although the incidence of bacterial meningitis is decreasing, its diagnosis is sometimes difficult because patients often present with symptoms other than the classic triad of meningitis symptoms, including fever, nuchal rigidity, and a change in mental status. Furthermore, a population-based cohort study of community-acquired bacterial meningitis reported that delayed administration of antimicrobial agents negatively impacts patients [4,5]. Thus, the infection severity and prognosis are of interest when treating patients with bacterial meningitis. Unfavorable outcome predictions in patients with bacterial meningitis have been reported to include impaired mental status on admission, seizures, and hypotension. The existing literature includes studies identifying the risk factors for unfavorable outcomes in patients with bacterial meningitis [5,6]. However, studies on Japanese people are relatively fewer than on Caucasians and other Asian populations [5-9]. Therefore, this study retrospectively evaluated the prognoses of patients diagnosed with community-acquired bacterial meningitis at Showa General Hospital, Japan, from January 2004 to December 2021 using the modified Rankin scale (mRs).

## Materials and methods

Between January 2004 and December 2021, 208 patients at Showa General Hospital had positive CSF cultures, and two neurologists clinically diagnosed six patients with meningitis. All protocols were approved by the Ethical Committee for Clinical Research at Showa General Hospital (approval number: REC-310), and patients were provided opt-out consent.

Patient activities were evaluated based on their medical records to determine if they could care for themselves 30 days after hospitalization. Next, they were classified using the mRs with scores ranging from 0 to 6. Patients were grouped as those with (i) favorable (mRs: 0-3) and (ii) unfavorable (mRs: 4-6) outcomes. For example, if a patient was discharged home without any sequelae, they were grouped as having favorable outcomes (mRs: 0-3).

All patients were administered empirical antibiotics (meropenem, vancomycin, ceftriaxone, or ampicillin) until bacterial culture and antibiotic susceptibility results were available, after which definitive therapy was initiated. Additionally, dexamethasone was administered as an adjuvant in 22 (56.4%) patients. Neurological or intensive care specialists performed these treatments in accordance with the treatment guidelines.

CSF and blood tests

A blood test and lumbar puncture were performed on all patients upon arrival at the hospital, and antibiotic administration was commenced as soon as possible. Blood tests included hematological, biochemical, and coagulation analyses, while CSF was collected for biochemical and hematological analyses. The blood and CSF were also submitted for microbiological analyses, including Gram staining and culture tests. Although not all patients had a history of diabetes mellitus (DM), those with hemoglobin A1c (HbA1c) levels ≥6.5% and random blood glucose levels ≥200 mg/dL were diagnosed with DM following the Japanese Clinical Practice Guideline for Diabetes 2019 [10]. C-reactive protein (CRP) levels were measured by latex agglutination turbidimetric immunoassay (N-assay LA CRP-T B type, Nittobo Medical, Tokyo, Japan), and elevated CRP levels were defined as higher than 0.5 mg/dL.

Imaging analyses

Cerebral infarction was assessed either by magnetic resonance imaging (MRI) or cranial computed tomography (CT) performed during hospitalization. Patients with (i) a combination of high signals in diffusion-weighted images and low signals in apparent diffusion coefficient maps on MRI or (ii) low-density areas observed in CT images during hospitalization were diagnosed with cerebral infarction. All patients underwent cranial CT, and three could not undergo MRI due to bad clinical statuses, the impossibility of maintaining them at rest, or other problems.

Statistical analyses

Based on previous research and pathophysiology, we compared relationships between outcome and predicting factors, that is, CSF glucose, CSF cell count, the number of days to initiate antibiotics, Glasgow coma scale (GCS) on admission, age, complication of cerebral infarction, DM status, and C-reactive protein (CRP). These analyses were performed using the Wilcoxon signed-rank sum test and chi-square test. As the range of values varied for each variable, the data were normalized, and logistic regression analysis was performed. In logistic regression analysis, we narrowed down variables as CSF glucose and GCS on admission, based on discussions with several clinicians and previous studies [7,8]. We narrowed down the variables because the number of patients was small. Statistical significance was set at p-values equal to 0.05, and all analyses were performed using R software (version 4.1.2).

## Results

**Figure 1:**
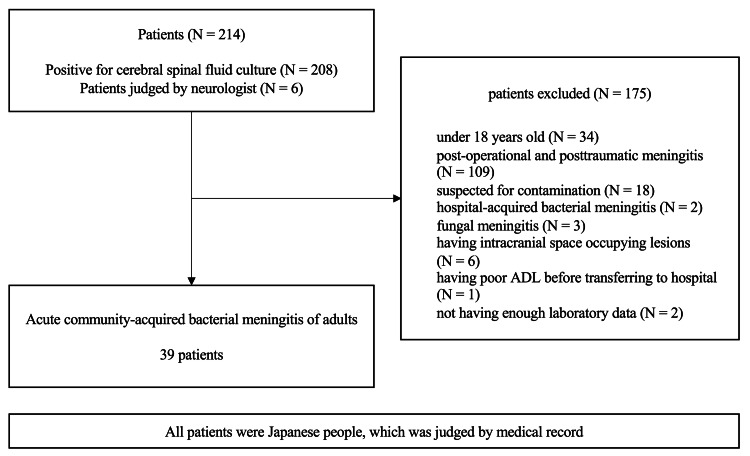
Selection of patients.

From a total of 214 patients, 175 were excluded for the following reasons: (i) 34 were <18 years old, (ii) 109 had postoperative and posttraumatic meningitis, (iii) 18 had suspected contamination, (iv) two had hospital-acquired bacterial meningitis, (v) three had fungal meningitis, (vi) six had occupying intracranial lesions, (vi) one had poor activities of daily living before being transferred to the hospital, and (vii) two had insufficient laboratory data. This study included 39 adult patients diagnosed with community-acquired bacterial meningitis (Figure 1, Table 1), of whom 15 (38.5%) were female, and 24 (61.5%) were male. All patients were Japanese, which was confirmed from the medical records. The median age was 64 years (range = 43-83 years), and the median CSF cell count was 2,308/μL3 (range = 20-22,880 /μL3). Causative microorganisms were detected in CSF samples from 33 (84.6%) patients via culture tests or Gram-staining. *Streptococcus pneumoniae* was the causative organism in 19 (48.7%) patients. The other isolated bacteria included *Streptococcus agalactiae* (N = 4, 10.3%), *Staphylococcus aureus* (N = 4, 10.3%), *Haemophilus influenzae* (N = 1, 0.03%), *Neisseria meningitis* (N = 1, 0.03%), *Serratia plymuthica* (N = 1, 0.03%), *Campylobacter fetus* (N = 1, 0.03%), methicillin-resistant *Staphylococcus aureus* (N = 1, 0.03%), *Staphylococcus capitis* (N = 1, 0.03%), *Streptococcus suis* (N = 1, 0.03%), and other unknown bacteria (N = 5, 12.8%). Blood cultures were positive in 23 (60.0%) patients.

**Table 1:** Characteristics of patients. mRs is a scale to present the severity of sequelae in patients, varying from 0 to 6, with 0 referring to normal status. GCS is a scale to present the consciousness level of patients, varying from 3 to 15, with 15 referring to a normal level of consciousness. SD = standard deviation; DM = diabetes mellitus; mRs = modified Rankin scale; GCS = Glasgow coma scale; CSF = cerebrospinal fluid; CRP = C-reactive protein

Characteristics	Number of patients (N = 39)
Patient characteristics	
Female, n (%)	15 (39)
Age, mean ± SD (years)	64 ± 13
Cerebral infraction, n (%)	15 (39)
DM, n (%)	5 (13)
mRs in 30 days <3, n (%)	19 (49)
GCS score at presentation, mean ± SD	11 ± 3
Laboratory features	
CSF	
CSF protein, mean ± SD (mg/dL)	460 ± 511
CSF cell count, mean ± SD (/3μL)	6530 ± 7721
CSF/blood glucose ratio, mean ± SD	0.2 ± 0.2
Blood	
CRP, mean ± SD (mg/dL)	18 ± 12

The median CRP level was 15.2 mg/dL (range = 0.15-44.57), and 19 (48.7%) patients had favorable outcomes (mRs: 0-3) after 30 days of hospitalization, while 20 (51.3%) had unfavorable outcomes (mRs: 4-6). Five patients had DM, including those previously diagnosed with DM and those newly diagnosed during hospitalization.

**Figure 2:**
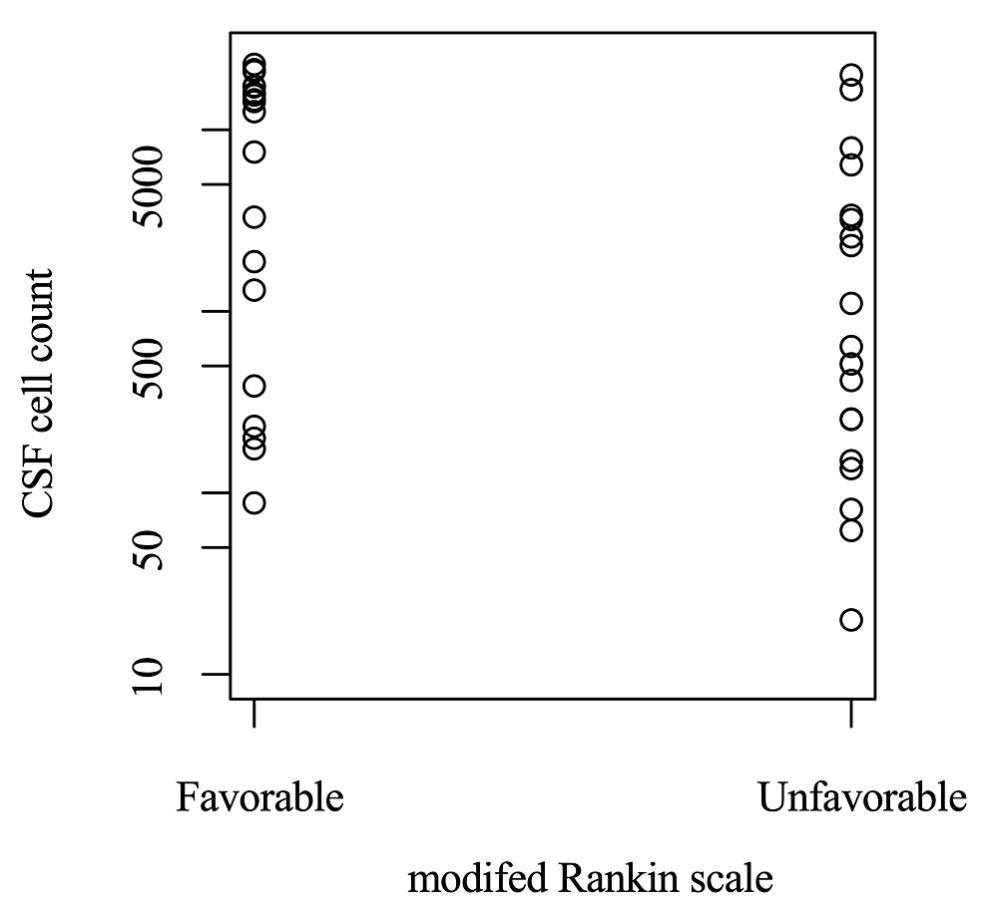
Relationships between CSF cell count and the modified Rankin scale. CSF  = cerebrospinal fluid

Five potential prognostic factors were associated with outcome and two of them had a strong association with outcomes on multivariable analysis. In non-parametric analysis, CSF cell count (p = 0.03), GCS on admission (p = 0.02), age (p = 0.005), a complication of cerebral infarction (p < 0.001), and CRP (p = 0.007) had relationships with poor prognosis (Table 2). On logistic regression analysis, CSF cell count was observed to be associated with poor prognoses (odds ratio (OR) = 0.86, 95% confidence interval (CI) 0.99995-0.99999, p = 0.0012) (Figure 2, Table 3). Moreover, GCS on admission was also observed to be associated with poor prognoses (OR = 0.93, 95% CI = 0.89145-0.97290, p = 0.0029) (Table 3).

**Table 2:** Relationships between outcome and predicting factors. CSF = cerebrospinal fluid; GCS = Glasgow coma scale; DM = diabetes mellitus; CRP = C-reactive protein

Predictive factor	P-value
CSF glucose	0.98
CSF cell count	0.026
The number of days to initiate antibiotics	0.47
GCS on admission	0.02
Age	0.0054
Cerebral infarction	<0.0010
DM	0.31
CRP	0.0067

**Table 3:** Independent risk factors for unfavorable factors. CI = Confidence interval; GCS = Glasgow coma scale; CSF = cerebrospinal fluid

Variable	Odds ratio	95% CI	P-value
CSF cell count	0.86	0.99995-0.99999	0.0012
GCS on admission	0.93	0.89145-0.97290	0.0029

## Discussion

Mortality rates of bacterial meningitis are still high due to the poor prognoses of patients with bacterial meningitis. In the United States, the mortality rate in adults is 16.4%, which increases with age; the mortality rate in patients between 18 and 34 years old is 8.9%, while the rate in patients >65 years old is 22.7% [5]. Focal abnormalities of the central nervous system, such as hemiparesis, aphasia, and gaze preference, are also observed in 16-28% of adult patients with bacterial meningitis [11,12]. The resulting damages are multifactorial, involving bacterial toxins, cytotoxic products of immunocompetent cells, and indirect pathology secondary to intracranial complications [13]. These abnormalities are mostly caused by cerebral edema, infarction, and hydrocephalus and severely affect patients’ quality of life. Moreover, these causes affect the consciousness of patients [14]. Cerebral infarctions observed in the patients in this study during MRI or CT performed after a few days of hospitalization were suspected to be caused by bacterial meningitis. It was difficult to assume that cerebral infarctions by lacunar, atheromatous, or cardiogenic and bacterial meningitis had occurred simultaneously. Cerebral infarction has been reported in patients with bacterial meningitis caused by *Streptococcus pneumoniae*, *Staphylococcus aureus*, and others [14,15].

Furthermore, this study observed a relationship between low CSF cell count and poor prognoses. One previous study reported that almost 2% of patients with community-acquired bacterial meningitis had completely normal initial CSF cell counts, although they did not necessarily have immunodeficiencies [16,17]. Repeated lumbar punctures showed that all patients with normal initial CSF cell counts had higher CSF cell counts than the initial values. The increased values were unrelated to poor prognoses; hence, CSF cell counts might have been measured before they became elevated. However, the authors of the study did not highlight this reason, and it did not include the completely normal CSF cell counts of patients with bacterial meningitis. Hence, their study did not identify the relationship between completely normal CSF cell counts and prognoses in patients with bacterial meningitis.

Additionally, Martijn et al. reported that CSF cell counts <1,000 cells/mm^3^ were significantly associated with unfavorable outcomes. Other studies also reported that low CSF cell counts were related to poor prognoses with outcomes such as paralysis caused by cerebral infarctions [5,8,18]. The present study also observed a similar trend in Japanese patients and could not identify the relationship between DM, CSF glucose, and the number of days to initiate antibiotics and poor prognoses.

On the other hand, some reviews have shown that granulocytes found in the CSF may imply an increase in neurotoxicity because leukocyte migration into the CSF may cause a blood-brain barrier injury and cerebral edema [13,19,20]. Considering these reviews, minor CSF cell count elevation might occur in cases of bacterial meningitis with low CSF cell counts. Lumbar punctures need to be repeated to observe the CSF cell count transition and confirm this. However, lumbar punctures are invasive and cause several complications. Hence, a repeated lumbar puncture to predict a patient’s prognosis is not a practical option, and additional studies and data are necessary.

This study had several limitations. First, it was a single-center study and the number of included cases was small. Second, data were retrospectively collected. Therefore, a multicenter prospective study with a larger sample size is necessary.

This study could not clarify the reason for low CSF cell counts in patients with bad prognoses. Nonetheless, the relationship between low CSF cell counts and poor prognoses was obvious based on the available data. Furthermore, this observed association between CSF cell counts and prognosis might aid in improving clinical decisions for patients with bacterial meningitis.

## Conclusions

This study observed a strong relationship between low CSF cell counts and poor prognoses. However, we did not observe any relationships between poor prognoses and age, CRP, and diabetes mellitus. As the study has some limitations, further research is needed.
